# Use of a Capsaicin Cough Challenge Test to Compare Four Different Techniques for Nebulization Delivery in Cats

**DOI:** 10.3390/vetsci11070320

**Published:** 2024-07-17

**Authors:** Jesús Talavera-López, Ana Tudela-González, Alberto Muñoz-Prieto

**Affiliations:** Department of Animal Medicine and Surgery, Veterinary School, University of Murcia, Campus de Espinardo nº 16, 30100 Murcia, Spainalberto.munoz@um.es (A.M.-P.)

**Keywords:** capsaicin, cough challenge, aerosol therapy, inhalation route, airway reactivity, feline

## Abstract

**Simple Summary:**

In people with respiratory diseases, treatment through inhalation is widely recommended and standardized. In veterinary medicine, few studies have investigated the effectiveness of treatments by the inhalation route. Among other factors, tolerance to the method used to administer the aerosol is key to its therapeutic success. This study has standardized a cough provocation test in cats using nebulized capsaicin at increasing concentrations and, through its use, compared the effectiveness and tolerance of the four methods most used in veterinary hospitals to administer oxygen and nebulized solutions. The results provide a cough challenge protocol that can be used in future studies for diagnostic and therapeutic purposes in cats with respiratory diseases. The protocol established that nebulization with a facial mask is the most effective, and nebulization in a chamber is the best tolerated. The flow-by method is a good alternative, but the hood method has poor cat tolerance.

**Abstract:**

Successful aerosol therapy might rely more heavily on proper drug delivery than on the effectiveness of the medication. This study compared four techniques for nebulization delivery in cats. Tolerance rate (TR) was subjectively evaluated (1–3). Increasing capsaicin concentrations were nebulized for objective evaluation of efficiency. The positive response (PR) was considered when more than five coughs were induced. The following delivery methods were tested: flow-by (FB); face mask (FM); plastic-covered Elizabethan collar (EC); and plexiglass chamber (PC). The number of PRs (NPR) and the concentration of capsaicin that induced a PR (CCP) were statistically compared (*p* < 0.05). The PC method was the best tolerated (TR = 3.0 ± 0.0), followed by the FB (2.7 ± 0.5) and FM (2.6 ± 0.5). The EC was very stressful, and the test had to be suspended in four out of nine cats. The lowest CCP was found with the FM (91.8 ± 157.2 µM), followed by the FB (166.7 ± 190.9 µM), PC (242.6 ± 244.8 µM), and EC (350.0 ± 225.7 µM), with significant differences only between the FM and EC (*p* = 0.02). The highest NPR (8) corresponded to the FM, followed by the FB (7), the PC (5), and the EC (3), with significant differences between the FM and EC (*p* = 0.016). In conclusion, the capsaicin cough test induces reproducible and quantifiable cat responses. The FM is the most efficient for nebulization delivery, offering good compliance and the best quantitative results. FB nebulization is less efficient but may be practical if the FM is not tolerated. PC offers minor efficiency but may be useful for very stressed or aggressive cats. The EC presents low efficiency and compliance.

## 1. Introduction

In veterinary medicine, respiratory diseases are commonly encountered in daily veterinary practice and often represent both a diagnostic and therapeutic challenge. In many of these diseases, especially in chronic diseases, the treatments administered by a systemic route do not always reach therapeutic concentrations in the target tissues, influenced by factors related to the metabolism, absorption, and penetration of drugs [1,2,3]. Also, the systemic administration of certain drugs (e.g., corticoids and aminoglycosides) may induce many potential systemic side effects that limit their therapeutic benefits [4,5]. The inhalation route has been used daily in human medicine for a long time to provide the local delivery of various medications to the airways [6,7,8,9]. Sterile saline nebulization is also commonly used in nursing care to favor airway hygiene and liquefy secretions in patients with respiratory diseases.

In recent years, a resurgent interest in inhaled therapies has emerged in veterinary medicine. An increasing number of clinical and experimental investigations have been published for cats [5,10,11,12,13,14], dogs [15,16,17,18,19], and horses [20,21,22,23,24]. Currently, despite the lack of standardized guidelines for the administration and dosage of inhaled medications in veterinary medicine, many experts advocate its use in treating a range of respiratory conditions [1,2,3,4,18,25,26]. Beyond its use for the treatment of respiratory diseases, the inhalation route (Lavender Essential Oil, for example) has shown usefulness in improving animal welfare and meat quality in farm animals [27]. Nevertheless, due in part to the inherent lack of cooperation of the animals, the use of this route is far less common than in human medicine. The lack of objective data on efficacy and tolerance is another factor that discourages many veterinarians from using inhaled medications. Recent studies have determined that 78% of board-certified specialists use prednisone/prednisolone as the first option for the treatment of chronic cough in dogs, and only 21% use inhaled fluticasone [28]. Many of them (50%) permit their use for long periods.

Effective therapy via the inhalation route is widely recognized to depend more on proper drug delivery than on the drug’s efficacy [3,7]. In many veterinary centers, in-hospital nebulization therapy is routinely used, but, because of the lack of standardized guidelines, the application of these treatments is based on the personal preferences or clinical experience of the veterinary practitioner. In human medicine, nebulization delivery to the patient is usually performed using a mouthpiece (adult patients) or a face mask (pediatric patients, patients who are in a life-threatening condition, or in-hospital treatments) [3,7]. The disposable methods to deliver the nebulized solution to the veterinary patient are analogous to those used for the administration of oxygen, including the flow-by method, face mask, the hood method (plastic-covered Elizabethan collar), and the cage method (plexiglass chamber, carrier, incubator, etc.). There are no concrete data on the comparative effectiveness and tolerance of these methods in cats.

The experimental induction of cough is an important component of clinical cough research [29,30]. Measurement of cough reflex sensitivity allows evaluation of the effect of pharmacological and other interventions on the cough reflex, as well as the performance of epidemiological studies relevant to cough. The most used tussive agents include capsaicin, citric acid, and ultrasonically nebulized distilled water (fog) [29,30]. Although these tests have been widely used in humans for more than 40 years and have been honed as useful epidemiological and pharmacological tools, there is very little information about them in veterinary medicine [29,30,31,32], and their use is not standardized.

The main aim of this study was to validate a cough challenge test in cats to compare the efficiency and compliance of four methods for nebulization delivery. The objectives of the study were: (1) to standardize a cough challenge test with capsaicin in cats; (2) to compare the efficacy of four methods for nebulization delivery in cats based on the differences in response to the capsaicin test; and (3) to subjectively compare the compliance of cats to the four methods of nebulization delivery.

## 2. Materials and Methods

### 2.1. Animals

Nine adult domestic short-hair laboratory cats of both sexes (5 male, 4 female) were used (mean age, 4.5 ± 0.6 years; mean body weight, 3.8 ± 0.5 kg). At inclusion, the cats were considered healthy based on the absence of clinical signs, normal physical examination, normal CBC count, normal serum biochemistry, and normal thoracic radiographs. The cats had no history of respiratory tract problems in the past 4 weeks. The cats were maintained in a controlled environment (without exposure to tobacco and smoke) and fed with a commercial dry diet.

Before the start of the nebulization studies, each cat was acclimated to each technique for nebulization delivery. This acclimation consisted of a short training session on each method during the first week of the study design. On different days, each cat was familiarized with each of the 4 methods by gentle repeated placement, first without the use of the nebulizer and, after several trials, with saline nebulization. All experiments were conducted according to general guidelines about the care and use of laboratory animals and under protocols approved by the Bioethics Commission of the University of Murcia, Spain (date of approval 26 October 2007) and executed by a veterinarian highly experienced in feline medicine.

### 2.2. Capsaicin Cough Challenge Test

Capsaicin powder (Sigma-Aldrich, Inc., Madrid (M), Spain) was dissolved in a physiologic saline solution to obtain a stock solution of 500 µM. This solution was further diluted to obtain working solutions of increasing concentrations (3, 10, 30, 60, 90, 100, and 300 µM). A pilot study using five healthy cats was first performed to establish the response ranges to design the definitive working protocol. No cat showed a positive response to concentrations lower than 3 µM, so this was used as the initial concentration for the rest of the challenges. Thus, for the challenge tests used in this study, capsaicin was nebulized using an ultrasonic nebulizer (OMN NE U07, Omron Healthcare Europe, Hoofddorp (NH), The Netherlands) in increasing concentrations (3, 10, 30, 60, 90, 100, 300, and 500 µM) by means of a dose–response protocol. Positive response (PR) was defined as the induction of more than five successive coughs (Video S1). Each concentration was nebulized for one minute, followed by a lasting period of one minute. If the cat showed a PR, the test was stopped, and the last concentration of capsaicin (CCP) was registered, as well as the number of coughs elicited (NC). If a positive response was not detected, the next concentration of capsaicin was nebulized. The same protocol was reproduced until the last concentration of capsaicin was reached (500 µM). If the cat did not show a PR at this concentration, the test was stopped, and the CCP registered was 500 µM.

### 2.3. Study Protocol

**Figure 1:**
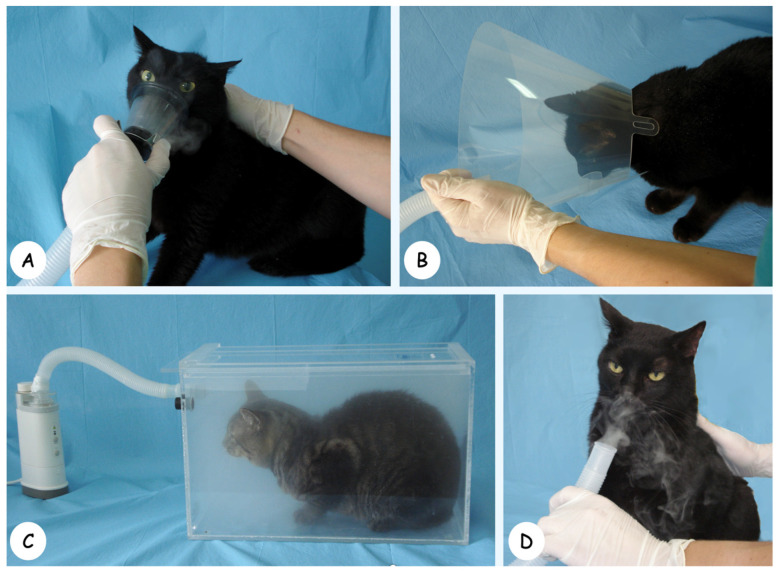
Photographs of the different techniques for nebulization delivery that were tested in this study. (**A**), nebulization using a face mask; (**B**), nebulization using a plastic-covered Elizabethan collar; (**C**), nebulization using a plexiglass chamber (18.5 L); (**D**), direct approximation of the nebulization outlet to the mouth/nose of the cat (flow-by method).

On separate days, the cough challenge described above was repeated in all cats, each time using a different nebulization delivery technique (Figure 1): (1) direct approximation of the nebulization outlet to the mouth/nose of the cat (flow-by method, FB); (2) using a feline anesthetic face mask (FM); (3) using a plastic-covered Elizabethan collar (EC); and (4) using a plexiglass chamber (18.5 L) (PC). The tests were always performed with the cats lightly sedated (acepromazine, 0.06 mg/kg + buprenorphine, 0.01 mg/kg, IM) to reduce stress and to standardize respiratory rate and depth. A washing period of at least one day between each test on the same cat was always respected.

Tolerance of each method was subjectively evaluated using the observation of signs of resistance or discomfort with each device and was classified as follows: (1) Excellent (three points): the animal remained calm during the test without signs of stress and needing no or minimal manual restraint; (2) Good (two points): mild signs of anxiety or discomfort at the beginning or during the test that required manual restraint but not influencing the development of the test; (3) Poor (one point): manifest and/or persistent signs of anxiety or discomfort that influenced the development of the test or even caused it to be stopped and restarted.

### 2.4. Statistical Analysis

A commercial software package (IBM SPSS Statistics 28.0.1.1 (14), Inc., Chicago, IL, USA, 2021) was used for statistical analysis. The data for the different study variables did not meet the criteria of normality (Shapiro–Wilk test) and were analyzed using non-parametric tests. The NPR, the CCP, and the NC elicited with each of the four techniques (FB, FM, EC, and PC), as well as the tolerance rate, were statistically compared using Chi-square, Friedman, and Wilcoxon tests for repeated measures. The Chi-square test was used to assess the independence between the categorical variables PR (yes/no) vs. delivery method (FB, FM, EC, and PC) in a contingency table under the null hypothesis of independence. The Friedman Test was used to compare the NC and the CCP between the 4 groups of the delivery method variable (FB, FM, EC, and PC). The Wilcoxon test was used to compare the NC and the CCP between each pair of the 4 groups of the delivery method variable (FB, FM, EC, and PC).

For all statistical analyses, statistical significance was assigned for values of *p* < 0.05.

## 3. Results

All cats enrolled in this study were responders to capsaicin. The PR (more than five successive coughs, Video S1) occurred at varying concentrations depending on each cat and according to the method employed. Usually, discomfort signs, salivation, and frequent swallows predicted the onset of coughing, but sometimes, they were the only manifestation even when the maximal concentration of capsaicin was nebulized (not PR).

When a PR was achieved, the intensity of the response was also variable. The mean NC was 21.13 ± 12.2 (10–53), without significant differences between methods (FM–EC, *p* = 0.109; FM–FB, *p* = 0.655; FM–PC, *p* = 0.285; FB–EC, *p* = 0.180; FB–PC, *p* = 0.655; EC–PC, *p* = 0.109). When the response was intense, the cats also showed mild respiratory distress with tachypnoea, which sometimes started before the PR was identified.

**Figure 2:**
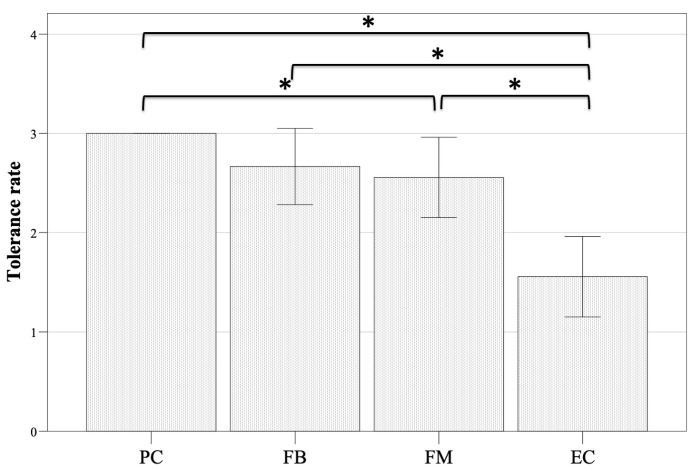
Mean ± 95% confidence intervals of the tolerance rate to four methods for nebulization delivery of increasing concentrations of capsaicin in 9 cats. PC, Plexiglass chamber; FB, flow-by; FM, face mask; EC, plastic-covered Elizabethan collar. * *p* < 0.05.

The PC technique was the best tolerated (tolerance rate, 3.0 ± 0.0; Figure 2). Upon immediate entry into the cage, cats typically exhibited escape attempts, often accompanied by vocalizations. However, after 1–3 min, they remained calm and motionless, although they occasionally engaged in position changes during the test, one even standing with her back to the output of the nebulizer. When this occurred, the test was not suspended but continued to the end. The FB and FM techniques also showed acceptable tolerance (2.67 ± 0.5 and 2.56 ± 0.53, respectively, *p* = 0.655; Figure 2). On tests with the FB method, most of the time, the cats did not require manual restraint (or they needed mild restraint just close to the point of PR), and they accepted with acquiescence the proximity of the nebulization tube. Conversely, during the FM test, manual restraint was frequently required, especially before/during coughing episodes (PR). So, the tolerance rate to the FM was significantly lower than that to the PC technique (*p* = 0.046). However, the EC technique was very stressful, and the test had to be suspended and repeated in 4 out of 9 cats (44%). Thus, the tolerance rate of the EC technique (1.56 ± 0.53) was significantly lower than each of the three other methods (FB, *p* = 0.015; FM, *p* = 0.007; PC, *p* = 0.006; Figure 2). The placement of the EC system was stressful, even before the nebulization procedure started.

**Figure 3:**
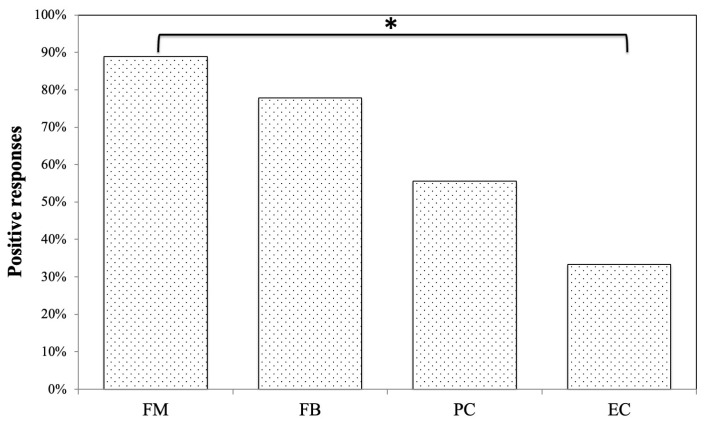
Percentage of positive responses (more than 5 coughs) to a capsaicin challenge test according to the use of four methods for nebulization delivery in 9 cats. FM, face mask; FB, flow-by; PC, Plexiglass chamber; EC, plastic-covered Elizabethan collar. * *p* < 0.05.

The FM technique induced positive responses in a greater number of cats (NPR = 8/9, 88.9%), followed by the FB technique (NPR = 7/9, 77.8%), the PC technique (NPR = 5/9, 55.6%) and the EC technique (NPR = 3/9, 33.3%). The differences were only statistically significant when comparing the FM and EC techniques (FM–EC, *p* = 0.016; FM–FB, *p* = 0.527; FM–PC, *p* = 0.114; FB–EC, *p* = 0.058; FB–PC, *p* = 0.317; EC–PC, *p* = 0.343) (Figure 3).

**Figure 4:**
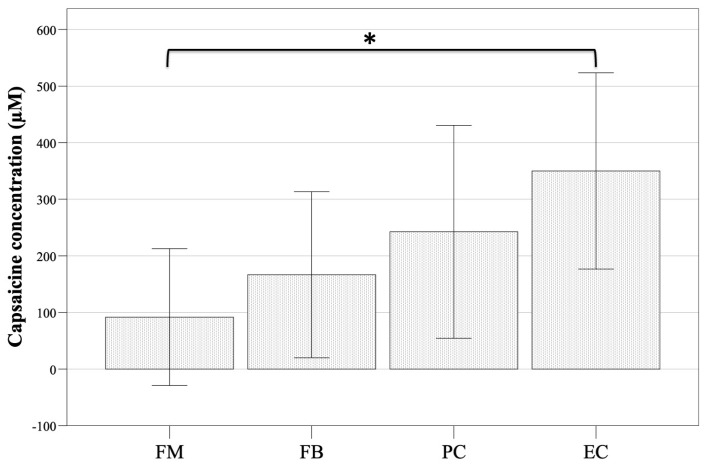
Mean ± 95% confidence intervals of the capsaicin concentration necessary to induce a positive response to a capsaicin challenge test according to the use of four methods for nebulization delivery in 9 cats. FM, face mask; FB, flow-by; PC, Plexiglass chamber; EC, plastic-covered Elizabethan collar. * *p* < 0.05.

The technique that induced a PR at a lower CCP was the FM (91.8 ± 157.2 µM), followed by the FB technique (166.7 ± 190.9 µM), the PC technique (242.6 ± 244.8 µM), and the EC technique (350.0 ± 225.7 µM), although the differences were only statistically significant when comparing the FM and EC techniques (FM–EC, *p* = 0.02; FM–FB, *p* = 0.122; FM–PC, *p* = 0.161; FB–EC, *p* = 0.116; FB–PC, *p* = 0.733; EC–PC, *p* = 0.138) (Figure 4).

## 4. Discussion

This study describes for the first time an inhalation cough challenge protocol with increasing concentrations of capsaicin in conscious cats. This challenge provoked an evident and easily quantifiable tussive response. Using this test, it has been possible to comparatively assess the efficiency of various systems for delivering a nebulized solution. These findings may have interesting clinical implications and could be relevant in future studies with experimental or clinical purposes.

Coughing is one of the most frequent symptoms prompting both human and veterinary patients to seek medical care [2,30]. The experimental induction of cough using inhaled provocative agents serves a vital role in clinical cough research by allowing the measurement of cough reflex sensitivity and its modulation by pharmacological agents and other interventions [29,30]. A variety of tussive agents have been used to induce cough, such as capsaicin, citric acid, tartaric acid, acetic acid, and ultrasonically nebulized distilled water (fog) [29,30]. Capsaicin is the pungent extract of peppers that produces burning and irritation when placed on the skin [28]. Capsaicin induces cough by stimulation of the transient receptor potential vanilloid-1 (TRPV-1) within small nerves, leading to the discharge of nerve fibers at low concentrations [29]. The capsaicin challenge test has been used in human patients for more than 40 years and has gained favor among investigators because of its ability to induce cough in a safe, dose-dependent, and reproducible manner [29,30]. However, references to its use in veterinary medicine are very scarce. In dogs, previous studies have established the difficulty of reproducibly inducing cough, both by physical and chemical methods, including the capsaicin tussive test [31]. The same capsaicin challenge test used in the present study was also tested without success by us in dogs, in which it was impossible to obtain positive responses even by nebulizing the maximum possible concentration of capsaicin. A study involving anesthetized cats with induced asthma utilized a capsaicin challenge and concluded that it did not seem to serve as an effective broncho-provocative in cats; however, the cough-inducing effect was not examined [32]. In the present study, all cats enrolled were cough-responders to capsaicin. The response (more than five successive coughs) was repetitive and occurred at varying concentrations depending on each cat and according to the delivery method employed. Considering that previous studies have established that nebulized capsaicin does not induce bronchospasm in cats [32], the cough response was most likely due to TRPV-1 stimulation. This aspect could have diagnostic interest since it could help differentiate cats with asthma from cats with chronic bronchitis or other causes of chronic cough without predominance of bronchospasm.

Previous studies have compared various methods of inhaled medication administration through the measurement of dynamic respiratory variables [23] or pulmonary deposition of radiopharmaceuticals [17,33]. One advantage of the capsaicin challenge test used in the present study is that the positive response is easily evident and does not require complex equipment for measuring respiratory variables, although the use of a cough monitor could help to specify better and size the type and intensity of the response. As a disadvantage that must be considered, the sensitivity to capsaicin in people is greater than in cats, so the test should preferably be carried out in an extraction hood or well-ventilated areas and with adequate protection for the operators to avoid discomfort and the cough response that can be produced in themselves. Therefore, the planning and development of future studies involving cough in cats using a challenge with nebulized capsaicin is warranted. These studies aim to determine if it is possible to differentiate subpopulations of cats in which bronchospasm is not a pathophysiological determinant of their disease, which could have diagnostic and therapeutic selection implications. Future research might also involve conducting the test after pre-treating cats with bronchodilators and/or antitussives to help in therapy selection. Furthermore, it would be convenient to design longitudinal studies that repeat the capsaicin test in cats receiving treatment to objectively validate its efficacy. Finally, epidemiological studies involving asymptomatic cats subjected to the capsaicin test could be conducted to explore its potential usefulness in the early detection of lower respiratory tract diseases.

In humans, inhaled therapy is the preferred route of respiratory care for patients with chronic obstructive pulmonary disease and asthma, as well as for inpatient treatment for several respiratory diseases such as bronchitis and pneumonia [6,7,8,9]. In veterinary medicine, the inhalation route is currently recommended by many specialists for treating various causes of chronic cough, such as lymphoplasmacytic rhinitis, chronic bronchitis, eosinophilic bronchitis, feline and equine asthmatic bronchitis, for antibiotic therapy in specific types of pneumonia, and for thinning secretions and facilitating elimination in patients with respiratory infections [1,2,3,4,5,14,15,18,19,20,22,24,25,26]. The blood–bronchial barrier limits the access of systemically administered drugs to the airway lumen and the cells lining the lower respiratory tract. To achieve drug penetration, high systemic doses are often required [3]. The inhaled route allows selective treatment of the lungs directly by achieving high drug concentrations in the airway while reducing systemic adverse effects by minimizing systemic drug levels [6]. Drugs administered by aerosol obviate absorption, bypass degradation in the gastrointestinal tract and liver, avoid detrimental effects on the gut flora, and allow the use of drugs that are not bioavailable when administered orally [3]. Inhaled β2-agonist bronchodilators produce a more rapid onset of action than oral delivery. Some drugs are only active with aerosol delivery (e.g., for asthma patients, cromolyn and ciclesonide; for cystic fibrosis patients, dornase alfa). Aerosol drug delivery is painless and often convenient. Nevertheless, selection of the appropriate device for individual patients is very important since it may jeopardize and restrict dramatically the efficacy of the treatment [7]. The findings of the present study offer tangible data that enhance the objective understanding required for the appropriate choice of delivery method for inhaled therapy in cats, which could potentially be extrapolated to other animal species.

The importance of this type of technique is well illustrated by the plethora of relevant publications in human medicine [6,8,9]. In veterinary practice, inhaled therapy is used in aerosolization or nebulization. Aerosolization using metered dose inhalers (MDI) is useful for chronic domiciliary treatments with corticosteroids and bronchodilators as adjuvant therapy or replacement for systemic medications in the treatment of chronic airway inflammatory diseases such as feline asthma and canine and feline chronic bronchitis, and of horses with recurrent airway obstruction [2,3,4,14,18,19,20,21]. In contrast, airway nebulization therapy is mainly used for in-patient treatment of acute or subacute respiratory problems, mainly of infectious origin (bronchopneumonia, acute tracheobronchitis, kennel cough complex, etc.) [1,2,25]. In these cases, the main therapeutic targets include the administration of physiologic saline for airway humidification and fluidification of respiratory secretions and/or the administration of some antibiotics (particularly aminoglycosides) [1,2,25]. As in humans, the correct selection of the method for inhaled therapy directly impacts the efficacy of the treatment. This study has been conducted to provide objective data to guide clinicians in the selection of the method for nebulization delivery. The FM technique induced positive responses in a greater number of cats and was also the method that induced a PR to the tussive test at a lower concentration of capsaicin compared with the rest. This result confirms that this method ensures the inspiration of a greater amount of the nebulized solution. Previous studies in which conscious untrained cats have been nebulized with a nebulized radiopharmaceutical agent using an FM have demonstrated the effectiveness of this method in pulmonary deposition of the radiopharmaceutical [34]. The method was well tolerated by the cats in the present study, so it would be the method of choice if therapeutic efficacy (understood as the administration of the largest dose in the shortest possible time) is to be prioritized. However, it is possible that in restless or aggressive cats the tolerance is lower than that found in this study. In those cases, the best alternative would be the PC method, which provided the highest tolerance ratio, although the efficiency was lower. This method is close to that which is sometimes recommended for home nebulization therapies and consists of carrying out the sessions with the patient placed inside a carrier covered by a towel [25]. Since the chances of nebulized leakage will be greater with this system than with the PC, it may be assumed that the effectiveness will be even lower, although it may be compensated by comfort. Previous studies using gentamicin nebulization in dogs have demonstrated the efficacy of this method (carrier covered by a towel) in the treatment of bronchial infections caused by *Bordetella bronchiseptica* [25].

This study may have several limitations. Placebo inhalations were not planned during the capsaicin challenge test. Protocols of a human cough challenge recommend that to increase challenge blindness, placebo inhalations of physiologic saline should be randomly interspersed between incremental concentrations of capsaicin to minimize or avoid voluntary suppression or conditioned responses in subjects anticipating progressively stronger concentrations of the tussive agent [30]. However, although cats can develop some degree of a conditioned response when tested repeatedly, it is unlikely that they would have developed conscious anticipatory behavior to influence the outcome of the test. Furthermore, the design of the test used in this study implies the cumulative effect of the succession of incremental concentrations, so intercalating saline solution could coincide with a response due to accumulation rather than conditioning of the animal. The succession of incremental concentrations at fixed nebulization times (one minute) and waiting times between doses (one minute) systematically provides a fixed protocol with comparable intra- and inter-individual responses. Future studies with placebo inhalations interspersed between capsaicin concentrations could clarify whether this aspect is relevant in the standardization of the test in veterinary medicine.

Another limitation is that this study did not evaluate drug deposition. The results of the capsaicin challenge are based on the evident effects (coughing spells) produced by capsaicin when nebulized with the same protocol but varying only in the method of delivery of the nebulized solution. It cannot be guaranteed that the respiratory strategy linked to the method itself was not distinct, or that other uncontrolled factors were introduced that could lead to differences in lung deposition, unrelated to the delivery method of the nebulized capsaicin solution. However, since it was the only variable that changed between each repetition and since the same animals were used to be compared with themselves, it is unlikely that these aspects, although having theoretical interest, are relevant as such in the validation of the test for this study. Conducting future studies with the nebulization of radiopharmaceuticals to compare the nebulized delivery methods used in the present study, based on the degree of deposition and distribution within the airways, could help confirm these aspects.

There could also be some limitations in this study when extrapolating the tussive test design in practice. This study was performed in healthy cats. The distribution of nebulized and capsaicin deposition might be altered in cats with respiratory disease, associated with changes in airflow dynamic because of impaired mucociliary clearance, mucous retention, and airway remodeling. These variables could determine a greater or lesser sensitivity to the test in cats with respiratory diseases that would require a new adjustment of the concentrations of capsaicin to be nebulized to determine differences between individuals. It is justified to conduct future studies in cats with respiratory diseases that allow the test to be extrapolated to the clinical setting, thereby exploring its diagnostic potential and aiding in the selection and monitoring of treatments.

## 5. Conclusions

The capsaicin cough challenge test used in this study induces reproducible and quantifiable cat responses. The FM is the most efficient technique for nebulization delivery in cats, offering good tolerance and the best quantitative results. FB nebulization is less efficient, but it may become practical if the FM is not tolerated. The PC technique offers a minor efficiency, but it may be useful for very stressed or aggressive cats. The EC technique presents low efficiency and tolerance.

## Data Availability

Data are contained within the article.

## Supplementary Materials

The following supporting information can be downloaded at: https://www.mdpi.com/article/10.3390/vetsci11070320/s1, Video S1: Positive response (more than five coughs) in a cat following nebulization of capsaicin by the cage method.
